# Pharmacological characterization of venoms from three theraphosid spiders: *Poecilotheria regalis*, *Ceratogyrus darlingi* and *Brachypelma epicureanum*

**DOI:** 10.1186/s40409-015-0017-8

**Published:** 2015-06-17

**Authors:** Alejandro García-Arredondo, Luis Rodríguez-Rios, Luis Fernando Díaz-Peña, Ricardo Vega-Ángeles

**Affiliations:** Laboratory of Chemical and Pharmacological Natural Product Research, School of Chemistry, Autonomous University of Querétaro (UAQ), Santiago de Querétaro, Querétaro Mexico

**Keywords:** *Poecilotheria regalis*, *Ceratogyrus darlingi*, *Brachypelma epicureanum*, Tarantula, Venom, Toxicity

## Abstract

**Background:**

Tarantulas (Theraphosidae) represent an important source of novel biologically active compounds that target a variety of ion channels and cell receptors in both insects and mammals. In this study, we evaluate and compare the pharmacological activity of venoms from three taxonomically different theraphosid spiders bred in captivity: *Poecilotheria regalis*, an aggressive arboreal tarantula from southeastern India; *Ceratogyrus darlingi*, an aggressive tarantula from southern Africa; and *Brachypelma epicureanum*, a docile tarantula from the Yucatan dry forest of Mexico. Prior to this study, no research had been conducted with regard to the composition and pharmacological activity of these venoms.

**Methods:**

The pharmacological characterization of the venoms was described for the first time by the assessment of their toxicity in crickets (LD_50_) along with their nociceptive (by using the formalin test), hyaluronidase, phospholipase A_2_, edematogenic and caseinolytic activity.

**Results:**

*P. regalis* and *B. epicureanum* venoms induced a similar lethal effect on crickets (LD_50_ = 5.23 ± 3.1 and 14.4 ± 5.0 μg protein/g 48 h post-injection, respectively), whereas *C. darlingi* venom (119.4 ± 29.5 μg protein/g 48 h post-injection) was significantly less lethal than the other two venoms. All three venoms induced similar edematogenic activity on rats but did not induce nociceptive behavior. The assessment of enzymatic activity indicated that *P. regalis* venom induces significantly higher hyaluronidase activity (27.6 ± 0.9 TRU/mg) than both *C. darlingi* (99.7 ± 1.9 TRU/mg) and *B. epicureanum* (99.6 ± 1.6 TRU/mg); these latter venoms did not display phospholipase A_2_ or caseinolytic activity.

**Conclusions:**

This study demonstrates that these theraphosid spiders of different habitats produce venoms with different activities. *P. regalis* venom displays a high level of hyaluronidase activity, which may be associated with its potentially medically significant bite.

## Background

The Theraphosidae family (phylum Arthropoda, class Arachnida, order Araneae, suborder Mygalomorphae) is a sizable group of large spiders that are also called tarantulas. Their venoms are mainly composed of peptides and proteins that target a variety of ion channels and cell receptors in both insects and mammals. Other components usually found in their venoms are nucleotides, free amino acids, neurotransmitters, polyamines and salts [1]. Due to the diverse mixture of components, tarantula venoms represent an important source of novel biologically active compounds that are potentially valuable for the development of therapeutic agents, tools for pharmacological research, and insecticidal peptides for potential use in agriculture [2–4].

To date, approximately 975 species of tarantulas (in 128 genera) have been recognized, which are found in several ecological niches worldwide, and it can be expected that many new species have yet to be described [5]. A total of 226 proteinaceous toxin sequences have been described to date from approximately 20 theraphosid species, the majority of which are small peptides that are ion-channel modulators [6]. Therefore, there is a significant interest in the study of the venoms of Theraphosidae species, not only because they constitute a potential source of valuable biomolecules, but also because they represent a large group that is relatively unexplored. In addition, the considerable size of these spiders permits easier venom extraction.

Tarantulas have become popular pets and the availability of several species has been facilitated by captive breeding. In this work, we focus on the pharmacological activity of the venoms from three taxonomically different theraphosid spiders widely kept and bred in captivity: two Old World tarantulas (*Poecilotheria regalis* and *Ceratogyrus darlingi*) and a new world tarantula (*Brachypelma epicureanum*). *P. regalis*, commonly referred to as the “Indian ornamental tree spider”, is an arboreal tarantula from southeastern India and it is considered aggressive and very fast. It is also one of the most popular tarantulas for collectors. *C. darlingi*, commonly called the African rear-horned Baboon, is an aggressive tarantula from southern Africa. Its venom is believed to be no more than mildly toxic to humans and it is the most common *Ceratogyrus* species held in captivity [5]. Finally, *B. epicureanum* is a tarantula from the Yucatan dry forest of Mexico and it is considered nonaggressive [5, 7].

In general, tarantulas are not harmful to humans and there is no record of human deaths resulting from a bite by these spiders [1, 8]. However, it is clear that some venoms are more toxic than others and may cause serious discomfort that might persist for several days. Some reports of tarantula bites suggest that the toxicity of Old World species is higher than that of the New World species, especially the members of the genus *Poecilotheria* [9, 10]. In a recent review of the literature on bites of species of this genus, it was found that a delayed onset of severe muscle cramps lasting for days is characteristic of *Poecilotheria* bites; other registered symptoms were local swelling, erythema, and moderate to severe pain [11].

Prior to this study, there had been no research conducted regarding the composition and pharmacological activity of the venoms of *P. regalis*, *C. darlingi*, and *B. epicureanum*. Thus, the aim of the current work was to provide quantitative information on the toxicology of these venoms in order to determine their pharmacological potential.

## Methods

### Reagents

Hyaluronic acid sodium salt from *Streptococcus equi*, hyaluronidase from bovine testes type IV-S, hexadecyltrimethylammonium bromide, protease from *Streptomyces griseus*, and carrageenan were purchased from Sigma (USA). Refined chemicals used for buffer preparations were purchased from J. T. Baker (USA). All reagents used in the determination of protein concentration and electrophoretic analysis were obtained from Bio-Rad (USA).

### Spiders

The adult female spiders (80–90 mm) bred in captivity used in this work were obtained from the Mexican Department of the Environment and Natural Resources-Management and Preservation Unit (SEMARNAT-UMA-IN-0062-JAL). All spiders were kept individually in plastic containers with water ad libitum and were fed weekly with crickets (*Acheta domestica*).

### Venom extraction

The spiders were immobilized by placing them in a small receptacle in a chloroform atmosphere for 15 min and then they were attached by the cephalothorax to a hand-made holder. The fangs were raised and positioned in a 1.5 mL microcentrifuge tube, ensuring that there was not any contamination with digestive fluids and saliva. Then, electrostimulation was applied with a pair of modified electrodes using an electrophoresis power supply (PowerPac Basic Power Supply Bio-Rad, USA) at 10–15 V. Electrodes were positioned within the fissure between the chelicerae and the cephalothorax, and stimulus was applied for two seconds with a three-second interval between shocks for 1 min. The collecting tubes were weighed before and after the venom extraction in order to estimate the weight of the liquid venom obtained. After the extraction, the venom was centrifuged and stored at −70 °C until it was used. Extraction was performed monthly in order to allow sufficient time for the tarantulas to replenish their venom supply. Analysis of venom profiles by electrophoresis was done individually and differences were not observed between specimens of the same species or between successive extractions from the same specimen. In this manner, venoms from six or seven extractions were used to form a pool of venom from each species for the biological assessments.

### Protein quantification

The protein content of the venoms was determined by the Bradford assay [12], using a standard curve prepared with lyophilized bovine serum albumin.

### Toxicity in crickets

The venom toxicity was assessed by determining the median lethal dose (LD_50_) in crickets (*Acheta domestica* purchased from Maskota SA de CV, Mexico) of undetermined sex weighing 190–210 mg by a previously described method [13]. Briefly, venoms were assessed by thoracic injection into crickets (*n* = 5, for each venom dose) at several doses (1, 3.2, 10, 31.6, 100, and 316 μg protein/mL). All venoms were dissolved in insect saline composed of 200 mM NaCl, 3.1 mM KCl, 5.4 mM CaCl_2_, 4 mM MgCl_2_, 2 mM NaHCO_3_, 0.1 mM Na_2_HPO_4_, pH 7.2. The injection volume for all crickets, including the controls that received insect saline, was 10 μL. Injections were performed using a 0.3 mL-gauge insulin syringe (B-D Ultra-Fine, Terumo Medical Corporation, USA). After the injection, the crickets were placed in small plastic containers with food and water. Mortality was scored at 24 and 48 h post-injection. The LD_50_ values were interpolated by fitting log dose–response curves using non-linear regression analysis in Prism 5.0 (GraphPad Software, Inc., USA) and reported as the mean ± SEM of three replicates.

### Formalin test

Acute neurogenic and chronic inflammatory nociception were measured using the rat paw formalin test as described by Dubuisson and Dennis [14] with some modifications. Three quantities of each venom (5, 10, and 20 μg protein/paw) were dissolved in 50 μL of sterile saline solution (0.9 % NaCl) and injected subcutaneously into the right dorsal hind paw of male Wistar rats weighing 100–120 *g* (*n* = 3/group). The positive control group received 50 μL of 2.5 % formalin, and the negative group received 50 μL of saline solution through the same route of administration. During the test, each rat was placed in a transparent glass container and nociceptive behavior was quantified by counting the time of licking, flinching, and lifting of the injected hind paw. Measurements were made in two phases: the first phase (neurogenic) was evaluated during the period from 0 to 10 min after injection, and the second phase (inflammatory) was from 10 to 50 min after injection.

### Edematogenic activity

To assess the edematogenic activity of venoms, rat paw edema was measured with a manual hydroplethysmometer as previously described [15]. After subplantar injection of 50 μL of venom (40 μg protein/paw) on the right hind paw of male Wistar rats weighing 100–120 *g* (*n* = 3/group), the rat paw edema was measured every 10 min in the first hour and every 30 min in the second hour. The positive control group received 100 μL of 1 % carrageenan solution prepared with distilled water, and the negative group received 50 μL of saline solution through the same route of administration.

### Enzymatic activity

The hyaluronidase activity was determined according to a turbidimetric method [16]. Briefly, different concentrations of venoms (1, 2.5, 5, 7.5, 10, 15, 20, and 25 μg protein/mL), diluted in 150 μL of buffer (0.2 M sodium acetate, pH 6.0, containing 0.15 M NaCl), were incubated with 100 μL of substrate (1 mg of hyaluronic acid sodium salt from *Streptococcus equi* in 1 mL of acetate buffer) at 37 °C for 15 min. After the incubation period, 1 mL of hexadecyltrimethylammonium (2.5 %) in 2 % NaOH was added to the samples. The resulting turbidity was read at 400 nm in a microplate spectrophotometer (Benchmark Plus, Bio-Rad, USA) after 30 min of incubation at room temperature. As the reference for hyaluronidase activity, hyaluronidase from bovine testes type IV-S was used at the same concentrations as the venoms. The enzymatic activity was expressed as mean ± SEM (*n* = 3) in turbidity reducing units (TRU)/mg. One unit of activity corresponded to the amount of enzyme that produced a 50 % reduction in turbidity caused by 0.1 mg of substrate under the conditions described above.

In order to detect possible phospholipase A_2_ activity in the venoms (10 μg of protein), a secretory colorimetric assay kit (Cayman Chemical, USA) was used. This assay uses the 1,2-dithio analogue of diheptanoyl phosphatidylcholine as substrate. Free thiols generated upon hydrolysis of the thioester bond at the sn-2 position by phospholipases were detected using DTNB [(5,5′-dithio-bis-(2-nitrobenzoic acid)]. Color changes were monitored by a Benchmark Plus microplate spectrophotometer (Bio-Rad, USA) at 414 nm by sampling every minute for ten minutes. For this colorimetric assay, a phospholipase A_2_ from bee venom was used as the control. Phospholipase A_2_ activity was expressed as μmol of hydrolyzed substrate/minute/mg of protein.

In addition, caseinolytic activity was assayed in order to detect possible protease activity in the venoms. It was assayed according to a previously described method [17]. Aliquots (0.4 mL) of 2 % casein in 0.2 M Tris–HCl buffer (pH 7.5) were incubated with different quantities of venom (1, 10, 20, 30, 50, and 100 μg protein/mL) at 37 °C for 30 min. The reaction was stopped by adding 1.5 mL of 0.44 M tricholoacetic acid and allowed to stand for 30 min. The mixture was centrifuged at 1500 × *g* for 15 min. An aliquot (1 mL) was mixed with 2.5 mL of 0.4 M sodium carbonate and 0.5 mL of 1:2 diluted Folin reagent, and the color developed was read at 660 nm. The reference for protease activity was a protease from *Streptomyces griseus*. Activity was expressed as μmol substrate/minute/mg of protein.

### SDS-Polyacrylamide Gel Electrophoresis (SDS-PAGE)

Electrophoresis was performed as previously described [18]. Samples were diluted 1:1 in a sample buffer (Bio-Rad, USA, Cat # 161–0737). Samples under reducing conditions were diluted 1:1 in a sample buffer containing β-mercaptoethanol and heated at 95 °C for 5 min. Then, 18 and 14 % polyacrylamide gels, loaded with 15 μg of protein, were electrophoresed at 120 V for 2 h at 4 °C, using Tris-Glycine buffer (25 mM Tris, 192 mM glycine, pH 8.3; Bio-Rad, USA, Cat # 161–0734). Protein bands were visualized using Coomasie brilliant blue R-250 staining solution (Bio-Rad, USA, Cat # 161–0437). Molecular masses were determined by comparison with a broad-range polypeptide standard (Bio-Rad, USA, Cat # 161–0318).

### Ethics committee approval

The animal utilization was approved by the Committee of Bioethics of the School of Medicine, UAQ.

## Results

### Venom yield

*B. epicureanum* specimens yielded more venom (14.7 ± 2.6 mg of liquid/spider; *n* = 6) than *P. regalis* (8.7 ± 1.1 mg of liquid/spider; *n* = 6) and *C. darlingi* (4.0 ± 0.1 mg of liquid/spider; *n* = 7) specimens. For *B. epicureanum* the protein concentration was 3.2 ± 0.3 % of the venom weight, while for *P. regalis* it was 5.9 ± 0.7 %, and 16.3 ± 2.4 % for *C. darlingi*.

### Toxicity on crickets

**Fig. 1:**
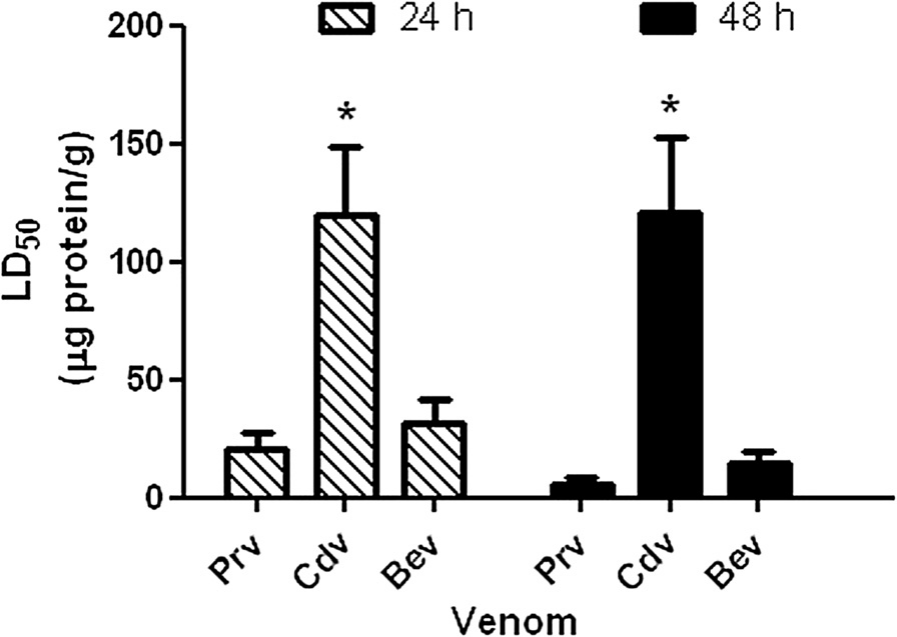
Comparison of LD_50_ values estimated from the injection of *P. regalis* (*Prv*), *C. darlingi* (*Cdv*), and *B. epicureanum* (*Bev*) venoms into crickets. Values represent the mean ± standard error (*n* = 3). *Significant difference (*p* < 0.05)

The results of the insecticidal activity on crickets showed that the LD_50_ values of the venoms of *P. regalis* and *B. epicureanum* were similar and the lethality of both venoms increased with time. The venom of *C. darlingi* was significantly less lethal than the other venoms (Table 1, Fig. 1). As for *P. regalis* and *B. epicureanum* venoms, it was observed that doses equal to or higher than 10 μg protein/g induced paralysis within 2 min, while doses equal to or higher than 31.6 μg protein/g of *C. darlingi* venom induced paralysis within 10 min. However, all crickets paralyzed with *C. darlingi* venom at 31.6 μg protein/g completely recovered after 24 h.

**Table 1:** LD_50_ values estimated from injection of *P. regalis*, *C. darlingi*, and *B. epicureanum* venoms into crickets

Venoms	Time post-injection	
	24 hours	48 hours
*P. regalis*	20.6 ± 6.2	5.23 ± 3.1
*C. darling*	119.4 ± 29.5	120.2 ± 32.3
*B. epicureanum*	31.5 ± 9.6	14.4 ± 5.0

Values represent the mean ± standard error (*n* = 3)

### Formalin test

**Fig. 2:**
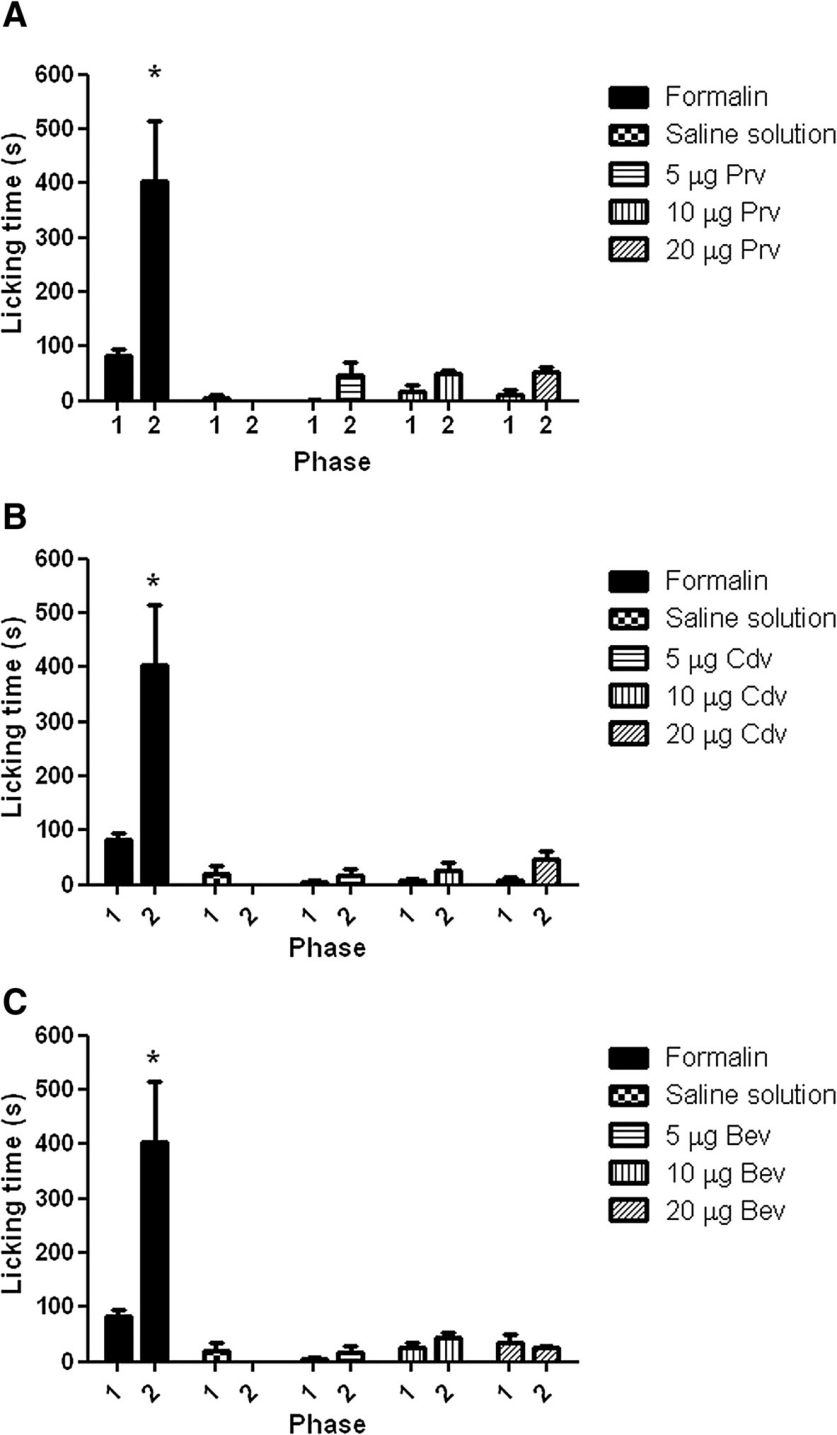
Formalin test for assessment of the nociceptive activity in rats of **a** *P. regalis*, **b** *C. darlingi*, and **c** *B. epicureanum* venoms at three different doses (5, 10, and 20 μg protein/paw). Nociceptive behavior in *phase 1* (0–10 min post-injection) and *phase 2* (10–50 min post-injection) was scored as the amount of time spent licking the injected paw. *Significant difference when compared with the negative control injected with saline solution (*p* < 0.05)

The results obtained with this test showed that in both phases (at doses of 5, 10, and 20 μg/rat hind-paw) the venoms of *P. regalis*, *C. darlingi*, and *B. epicureanum* did not induce nociceptive behavior in rats when compared to the negative control (saline solution). On the contrary, the formalin group was significantly different from all experimental groups and the negative control in the first and second phases (Fig. 2).

### Edematogenic activity

**Fig. 3:**
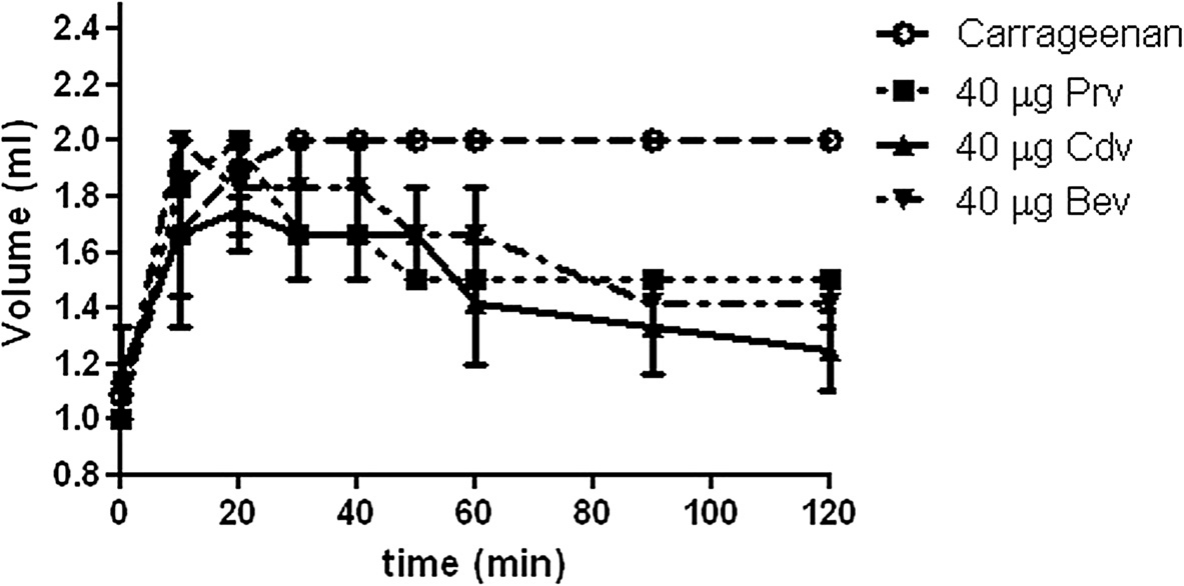
Volume of rat paw edema induced by subplantar injection of 40 μg of *P. regalis* (*Prv*), *C. darlingi* (*Cdv*), and *B. epicureanum* (*Bev*) venom protein. The positive control group received 100 μL of 1 % carrageenan solution, while the negative control group received 50 μL of saline solution (this did not induce detectable volume changes). Values represent the mean ± standard error (*n* = 3)

Assessment of the venoms’ edematogenic activity by subplantar injection of 40 μg of protein/rat showed that they induce a similar time-dependent increase in paw volume (Fig. 3). The maximum responses were observed at 10 min after administration, decreasing at approximately 60 min. However, *P. regalis* venom induced an evident redness immediately after administration. Carrageenan, used as a positive control, induced an increase in paw volume similar to that induced by the venoms but it did not decrease during the experiment. The negative control (50 μL of saline solution) did not induce a detectable response.

### Enzymatic activity

**Fig. 4:**
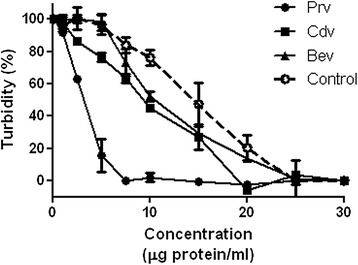
Comparison of hyaluronidase activity displayed in *P. regalis* (*Prv*), *C. darlingi* (*Cdv*), and *B. epicureanum* (*Bev*) venoms. Hyaluronidase from bovine testes type IV-S was used as a positive control. Values represent the mean ± standard error (*n* = 3)

The hyaluronidase activity of *P. regalis* venom (27.6 ± 0.9 TRU/mg) was significantly higher than that of *C. darlingi* (99.7 ± 1.9 TRU/mg) and *B. epicureanum* (99.6 ± 1.6 TRU/mg). The hyaluronidase from bovine testes type IV-S, used as a control, induced an enzymatic activity of 149.5 ± 1.4 TRU/mg under these conditions. The venom of *P. regalis* reached maximum activity at a concentration of 7.5 μg/mL, while the other venoms showed maximum activity between 20 and 25 μg/mL (Fig. 4). As expected, it was found that the venoms do not display phospholipase A_2_ or caseinolytic activity. In these assays, a phospholipase A_2_ from bee venom displayed an activity of 3.18 ± 1.0 μmol/minute/mg and a protease from *Streptomyces griseus* displayed an activity of 980.4 ± 5.2 μmol/minute/mg.

### SDS-PAGE analysis

**Fig. 5:**
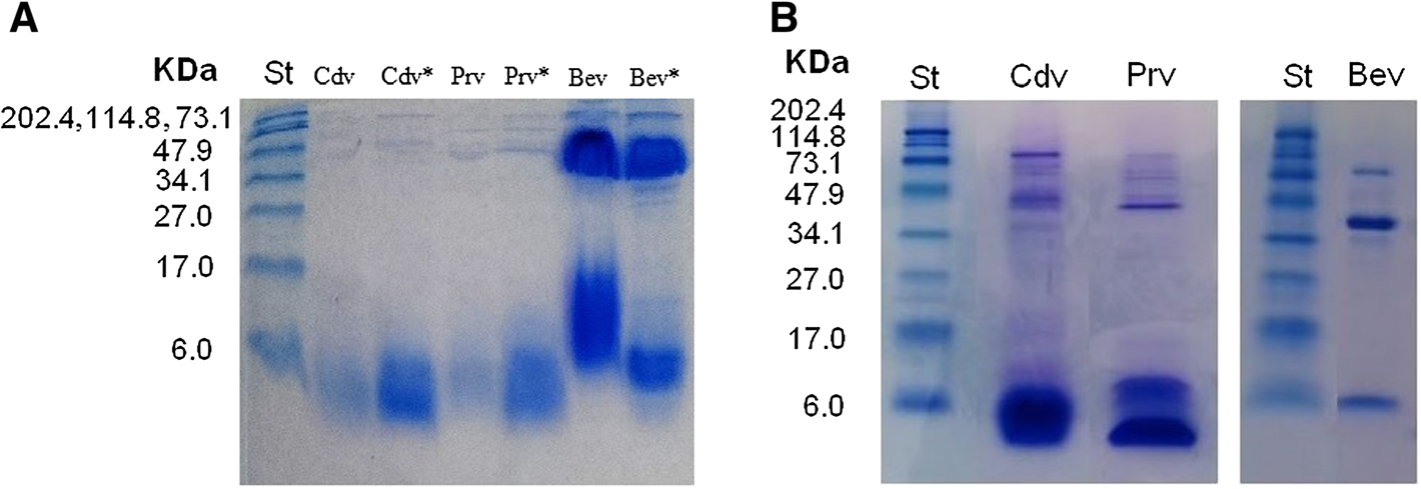
**a**. SDS-PAGE gel (18 % acrylamide) showing the protein profiles of *P. regalis* (*Prv*), *C. darlingi* (*Cdv*) and *B. epicureanum* (*Bev*) venoms. Prv*, Cdv* and Bev* correspond to the venoms under reducing conditions. **b**. SDS-PAGE gel (14 % acrylamide) showing the protein profiles of the venoms under reducing conditions. The protein profiles were compared with a broad-range polypeptide standard (*St*), and the masses of the molecular weight markers (in kDa) are shown on the *left* of the gels. Protein bands were visualized with *Coomasie blue*

Analysis by electrophoresis provides a preliminary overview of the proteins present in the venoms. Cdv and Prv profiles do not show important differences under reducing and not reducing conditions, and it is evident that these venoms contain bands in two main regions, representing two ranges of mass: 2–15 kDa and ~40 to ~100 kDa. In contrast, Bev profile evidently change under reducing conditions (Fig. 5a).

## Discussion

The relative toxicity of tarantula venoms is extremely variable. These spiders’ typical prey mainly consists of insects and other arthropods, but the venoms from some species have been reported to be lethal to pigeons, guinea pigs, rabbits, mice, rats, amphibians, snakes, lizards, and dogs [1, 8]. It is important to consider that the venom of each tarantula contains a specific mixture of components that bind to targets and may vary considerably, which was probably an evolutionary outcome that depended on the diversity of prey species in their environment. A comparative study on mice, using the venoms of 55 theraphosid spiders from several geographic areas, produced results that suggest an apparent higher toxicity of venoms from arboreal genera such as *Heteroscodra* (native to Africa), *Stromatopelma* (native to Africa), and *Poecilotheria* (native to Sri Lanka and India) [1].

In this study, we found that *P. regalis* venom was slightly more lethal to crickets than that of *B. epicureanum* and significantly more lethal than *C. darlingi* venom. The higher toxicity of *P. regalis* venom could be an adaptive advantage used to rapidly paralyze and kill prey in an aerial environment. The fact that the venom from the Mexican tarantula was more effective in killing crickets than the venom from the aggressive African tarantula is consistent with other studies. For example, it was found that the venoms from the Mexican tarantulas *Brachypelma harmorii* and *B. albiceps* were slightly more lethal to crickets than the venoms of several Australian tarantulas [19].

An interesting observation in the assessment of the insecticidal activity of these venoms was the capacity of *C. darlingi* venom to induce reversible paralysis. It is well known that several venomous animals produce toxins that modulate the activity of ion channels in order to capture their prey. Tarantula venoms include peptide neurotoxins that consist of 33–41 amino acid residues with three disulfide bridges that form an inhibitor cystine knot motif, defined as an antiparallel β-sheet stabilized by a cystine knot [1, 6, 20]. Many of these neurotoxins work together to provoke a characteristic paralysis on several types of prey.

At present, 27 of the 95 neurotoxins from tarantula venoms listed in ArachnoServer target voltage-gated sodium (Nav) channels, most of them acting by inhibiting inactivation. In fact, three of these toxins were found in the venom of the African tarantula *Ceratogyrus marshalli*: β-theraphotoxin-Cm1a and β-theraphotoxin-Cm1b modulate several subtypes of Nav channels (Nav1.1, Nav1.2, Nav1.4, Nav1.5, and Nav1.8) by shifting the voltage dependence of channel activation to more depolarized potentials and by blocking the inward component of the sodium current, while β-theraphotoxin-Cm2a modulates Nav1.5 and Nav1.8 channels [6]. A more potent immobilization is promoted by the potassium channel blockers through the prevention of nerve repolarization; 43 of these toxins have been reported in ArachnoServer. Moreover, 36 tarantula toxins have been reported to be irreversible calcium channel blockers. Some of these toxins can target different types of channels (Nav, Kv, and/or Cav) and appear to function through a similar mechanism; they are known as promiscuous toxins [1, 6].

The complexity of tarantula venoms results in a synergistic action that may produce fast paralysis and death in prey. Therefore, it is probable that the mixture of neurotoxins contained in the venom of *C. darlingi* is not capable of inducing a fast and permanent paralysis like that of *P. regalis*. Some tarantula neurotoxins induce reversible paralysis in specific animal models. For example, U1-theraphotoxin-Cv1a, found in the venom of *Coremiocnemis valida* (Singapore brown tarantula), induces reversible paralysis in crickets, but not in cockroaches and mice [6]; and huwentoxin-V, purified from the venom of *Selenocosmia huwena* (Chinese bird spider), can reversibly paralyze locusts and cockroaches for several hours [21].

Despite the fact that theraphosid spider bites are considered to be harmless to humans, there is evidence to support the idea that bites of *Poecilotheria* spp. are of medical importance [1, 8, 10, 11]. In a recent literature review of bite reports of *Poecilotheria* spp., most of them referred to *P. regalis*. Symptoms included local swelling, erythema, and moderate to severe pain. Of these bites, 58 % caused generalized muscle cramps that began on average 10 h after the incident and persisted for 1–14 days. Further symptoms were burning sensation, heat, fever, myalgia, heavy breathing, increased heart rate, and even brief loss of consciousness [11]. A possible explanation for the higher toxicity of the *Poecilotheria* species in humans is associated with the quantity of venom injected.

In a comparative study, the quantity of venom milked from several theraphosid species was compared and it was observed that an average *Poecilotheria* species yields approximately 12 μL more venom than other theraphosids [22]. In the present study, the weight of milked venom was measured and it was observed that *B. epicureanum* specimens yielded on average approximately two-fold more venom than *P. regalis* specimens, while *C. darlingi* specimens yielded four-fold less venom than *B. epicureanum* specimens. However, there was no significant difference in the amount of protein contained in the venom milked from the three species. It seems that the protein content is similar, but it is more concentrated in *C. darlingi* venoms. Thus, it is quite possible that the toxicity of *P. regalis* venom is due to the mixture of toxins rather than to the quantity of venom injected.

Many components of animal venoms are used defensively to ward off predators or competitors by inflicting pain [23]. Depending on the species, tarantula bite victims experience moderate or severe pain [10, 11]. This variable symptom may be attributable to a combination of a mechanical injury caused by the spider’s large fangs, a slightly acidic pH (usually close to 5), and the presence of some components such as biogenic amines (serotonin and histamine), adenosine, adenosine triphosphate, and inhibitor cystine knot peptides (vanillotoxins and DkTx), which specifically activate the noxious heat-sensing transient receptor potentiating (TRP) V1 receptor that is also the target of capsaicin, the painful toxin in ‘hot’ chili peppers [1, 23–25].

The formalin test is widely used to evaluate inflammatory and non-inflammatory pain in rats [14]. The first phase of the test is believed to result from the direct activation of primary afferent sensory neurons; this response is associated with direct activation of TRP channels present in nociceptors [26]. It has been proposed that the second phase reflects the combined effects of TRP channel activation and the development of an inflammatory response that prolongs the pain triggered by mediators such as interleukins 1β, 6, and 8, TNF-α, eicosanoids, and nitric oxide [27, 28]. In this study, the results obtained in the formalin test indicated that the *P. regalis*, *C. darlingi*, and *B. epicureanum* venoms did not produce a significant nociceptive effect at the tested doses. However, all three venoms tested in this study induced inflammatory responses. Similar results were observed with the venom of the Brazilian theraphosid spider *Acanthoscurria paulensis* [29]. The weak nociceptive responses observed with these venoms may be due to the presence of analgesic toxins.

In contrast with theraphosid toxins that induce pain by activating TRPV1 receptors, there are other theraphosid toxins that have analgesic properties. For example, μ-TRTX-Hhn1b, isolated from the venom of *Ornithoctonus hainana*, and huwentoxin-IV, isolated from the venom of *Ornithoctonus huwena*, are Nav1.7 channel inhibitors that efficiently alleviate acute inflammatory pain and chronic neuropathic pain in animals [30, 31]. Mechanotoxin 4, isolated from *Grammostola rosea* venom, reduces mechanical and neuropathic pain by blocking stretch-activated cation channels [32]. Psalmotoxin 1, isolated from the venom of *Psalmopoeus cambridgei*, has very potent analgesic properties against thermal, mechanical, chemical, inflammatory, and neuropathic pain in rodents by blocking acid-sensing ion channel 1a; this results in the activation of the enkephalin pathway [33]. Protoxin-I, obtained from the venom of *Thrixopelma pruriens*, is the first known peptide antagonist of the nociceptor ion channel TRPA1 [34].

Enzymes are important components of the venoms of several animals, and hyaluronidase activity is widely displayed in spider venoms [35]. The substrate for hyaluronidases is the hyaluronic acid, a mucopolysaccharide that is the major constituent of the extracellular matrix [36]. It is believed that the presence of these enzymes in animal venoms helps to distribute other venom components by hydrolyzing connective tissue [35]. In this study, we found that all three venoms induce hyaluronidase activity. An important observation is that *P. regalis* venom induces significantly higher hyaluronidase activity, which could be related to its higher toxicity. We also found that the venoms do not display phospholipase A_2_ or caseinolytic activity, in agreement with previous studies that suggest that neither phospholipase A nor protease activity is present in tarantula venoms [35, 37].

Finally, analysis by electrophoresis reveals that the *C. darlingi* and *P. regalis* venoms contain bands in two regions in different proportions. One region consists of small peptides (2–15 kDa) that are mainly neurotoxins [1]. The other region consists of proteins with masses between 40 and 100 kDa comprising hyaluronidases (39–43 kDa) and other components [38–40]. The profiles of the venoms showed important differences in their compositions, mainly in the venom of *B. epicureanum*, in which it can be observed that the profile of this venom change under reducing conditions. However, further proteomic analysis is necessary to improve these differences.

## Conclusions

This study demonstrates that these theraphosid spiders of different habitats produce venoms with diverse activities. It is observed that *P. regalis* venom displays a high level of hyaluronidase activity, which may be associated with its potentially medically significant bite.
